# Two-Year Results of the Banded Versus Non-banded Re-sleeve Gastrectomy as a Secondary Weight Loss Procedure After the Failure of Primary Sleeve Gastrectomy: a Randomized Controlled Trial

**DOI:** 10.1007/s11695-023-06598-z

**Published:** 2023-05-09

**Authors:** Mohamed Hany, Mohamed Ibrahim, Ahmed Zidan, Ann Samy Shafiq Agayaby, Moustafa R. Aboelsoud, Muhammad Gaballah, Bart Torensma

**Affiliations:** 1 Department of Surgery, Medical Research Institute, Alexandria University, 165 Horreya Avenue, Hadara, Alexandria, 21561 Egypt; 2 Madina Women’s Hospital, Alexandria, Egypt; 3 Leiden University Medical Center (LUMC), Leiden, The Netherlands

**Keywords:** Revision surgery, Banded sleeve gastrectomy, Non-banded sleeve gastrectomy, Food tolerance, Stomach volume

## Abstract

**Background:**

Insufficient weight loss or weight regain has been reported in up to 30% of patients after laparoscopic sleeve gastrectomy (LSG). Approximately 4.5% of patients who undergo LSG need revisional surgery for a dilated sleeve.

**Methods:**

This randomized controlled trial compared the outcomes between banded (BLSG) and non-banded re-LSG (NBLSG) after weight regain. Percentage excess body weight loss (%EWL), percentage total weight loss (%TWL), associated medical problems, gastric volume measurement, and endoscopy were measured preoperatively and 1 and 2 years postoperatively.

**Results:**

Both groups (25 patients each) achieved similar % EWL and %TWL at six months, one year, and two years postoperatively (%EWL 46.9 vs. 43.6, 83.7 vs. 86.3, and 85.7 vs. 83.9) (*p*= > 0.151) (%TWL 23.9 vs. 21.8, 43.1 vs .43.3, 44.2 vs. 42.2) (*p*=>0.342), respectively. However, the body mass index was significantly lower with BLSG (24.9 vs. NBLSG, 26.9). Both groups showed a significant reduction in stomach volume after two years (BLSG -248.4 mL vs. NBLSG -215.8 mL). Food tolerance (FT) scores were significantly reduced in both groups, whereby BSLG had significantly lower FT with an average of -1.1 point. No significant differences were observed regarding improvement of the associated medical problems after the first and two years after revisional LSG or the postoperative complications between both groups.

**Conclusion:**

Laparoscopic re-LSG is feasible and safe with satisfactory outcomes in patients with weight regain after LSG who have gastric dilatation without reflux esophagitis. Both groups had comparable significant weight loss effects and improvement of associated medical problems. The BLSG tends to have a more stable weight loss after two years with a significantly lower BMI, lower stomach volume, and less weight regain. Food tolerance decreased in both groups but reduced more in the BLSG group. After a 2-year follow-up, we may regard both procedures are safe, with no significant differences in the occurrence of complications and nutritional deficits.

**Graphical Abstract:**

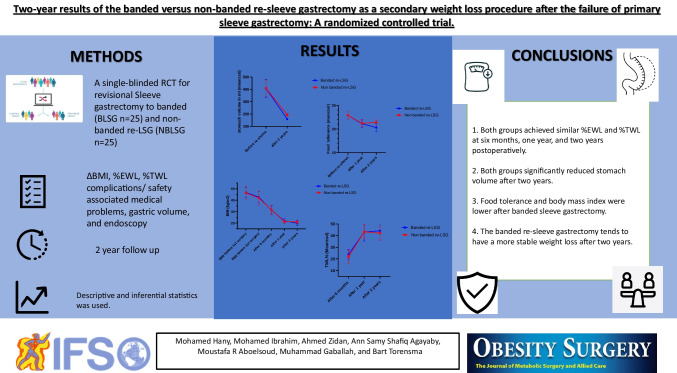

## Introduction

Laparoscopic sleeve gastrectomy (LSG) remains the most common bariatric metabolic surgery procedure worldwide, according to the fourth international federation for the surgery of obesity and metabolic disorders global registry report in 2018 [1]. Moreover, LSG is considered safer, has good outcomes, and lower complications and re-intervention rates compared to Roux en-Y gastric bypass (RYGB) [2, 3].

Nevertheless, insufficient weight loss or weight regain (WR) has been reported in up to 30% of patients after LSG [4, 5]. A systematic review by El Ansari et al. could categorize five reasons for weight regain or insufficient weight loss: hormonal/metabolic, dietary non-adherence, physical inactivity, anatomical surgical failure, and mental health [6].

When we look at sleeve dilatation, this may be an essential cause of WR because of the loss of restriction over time, which allows more food intake [6, 7]. The incidence of revisional surgery after weight regain has been reported to be 13% more frequent after LSG than after RYGB [2, 4, 6], and around 4.5% of patients undergo revisional LSG because of a dilated sleeve [7]. Moreover, in general, more than 61% of the patients have a dilation of that sleeve one year after primary LSG [8].

What is often considered for revision surgery in WR after LSG is conversion to RYGB, conversion to biliopancreatic diversion with duodenal switch, or conversion to single anastomosis duodenal-ileal bypass. Nevertheless, several studies have found that revisional re-LSG after weight regain following primary LSG is a good option that may achieve effective sufficient weight loss and improve associated medical problems [9-11].

To stimulate weight loss after bariatric-metabolic surgery, a banded laparoscopic sleeve gastrectomy (BLSG) can be initiated, which involves inserting a band around the upper part of the sleeve pouch to prevent its expansion and maintain better weight loss [12].

For patients with weight regain and a large stomach volume, laparoscopic re-sleeve (re-LSG) can be considered a revisional option with good secondary results regarding weight loss [13].

Some studies reported significantly higher weight loss in the BLSG cohort than in the non-banded LSG (NBLSG) cohort. Others reported that weight loss was comparable after BLSG or after RYGB [14-16]. However, other studies revealed no significant differences between BLSG and LSG regarding weight loss [17]. Moreover, patients who underwent BLSG are reported to have a higher incidence of eating problems with more regurgitation, vomiting, and dysphagia than those who underwent NBLSG because of the restrictive function of the band [14, 16, 18]. Thus, the effect of BLSG on the outcome of weight loss remains a subject for new research.

Sleeve volume by radiological assessment is a useful tool to report sleeve dilatation. This volumetric assessment can correlate dilatation and weight loss after LSG and BLSG [19]. Our clinic has experienced a rise in demand from patients with weight regain or insufficient weight loss who are hesitant to undergo revisional surgery other than LSG after comprehensive explanations of various BMS to all patients. Currently, no published randomized controlled trials have compared this. Therefore, our study aims to investigate the effectiveness of re-LSG with BLSG and NBLSG to compare the outcomes of weight loss, stomach volumes after re-LSG, food tolerance, the occurrence of complications, improvement of associated medical problems, and nutritional assessment over two years of the follow-up period.

## Methods

This randomized controlled trial aimed to compare the outcomes and two-year follow-up results of BLSG and NBLSG after weight regain following primary LSG. Recruitment occurred between March 2020 and mid-October 2020 at the Medical Research Institute, Alexandria University, Egypt, when the clinic was closed due to the COVID-19 pandemic. After re-opening the clinic, the patients were operated on between mid-October 2020- and mid-December 2020. The study protocol was approved and registered with the Ethics Committee by an institutional review board.

Before enrolling in the trial, patients were informed about the potential benefits and risks of both banded and non-banded procedures. Additionally, all participants were informed that the institute would cover the cost of band placement but that some would not receive the band during the procedure and would remain blinded until the end of the two-year follow-up period. Informed consent forms were signed by all participants prior to their involvement in the trial.

The revision procedures were performed at a specialized bariatric center. Two main/primary surgeons performed all the procedures with four assistant surgeons. Both main/primary surgeons performed BLSG and NBLSG procedures; all the cases were referred from other centers.

### Preoperative and Follow-Up During the Coronavirus Disease 2019 Pandemic

First, all patients were fully vaccinated (double dose of mRNA) and had a negative PCR (polymerase chain reaction) test result during bariatric metabolic revisional surgery.

Second, a combination of online virtual (when possible) and physical (with special time slots so that only one patient was present) was conducted during the preoperative and follow-up phases.

### Study Endpoints

Primary endpoints were percentage excess body weight loss (%EWL), percentage total weight loss (%TWL), change in BMI, and associated medical problems after the weight loss at one and two years postoperatively.

Secondary endpoints were gastric volume measurement and esophagogastroduodenal (EGD) transit gastroscopy one year postoperatively. EGD was only done on indication in year two.

### Multi-detector Computed Tomography

The sleeve volume was assessed preoperatively and two years after surgery using multi-detector computed tomography (MDCT) virtual gastroscopy and 3D reconstruction. MDCT gastrography was performed using multi-detector (64 detectors) CT scanners (Siemens SOMATOM® Perspective, Siemens Medical Solutions, Malvern, PA, USA). Patients were instructed to fast for at least 4 h before examination and were given an intravenous injection of 40 mg butyl-scopolamine, then asked to swallow 2 to 4 packs of effervescent granules (sodium bicarbonate) as tolerated on the table with no water [19] (Appendix 2).

### Surgical Techniques

Five standard ports were used, including three 12-mm ports (for the camera, right and left working ports) and two 5-mm ports (for liver retraction and the assistant). Pneumo-peritoneum was created after using optical trocars for entry while paying attention to the presence of adhesions from previous surgery. Dissection of adhesions around the gastric sleeve was performed using the energy device EnSeal® (Ethicon Endo-Surgery, Cincinnati, OH, USA). During reversion LSG, the stomach was resected 6 cm from the pylorus using a 40 French bougie with five standard ports. BLSG and NBLSG were reinforced and oversewed using absorbable 3/0 v-loc-sutures (Covidien, Mansfield, MA, USA).

For the BLSG group, peri gastric dissection was performed 4–5 cm from the gastroesophageal junction, and a size 7.5 (1.75 cm internal diameter) MiniMizer Gastric Ring® (Bariatric Solutions International, Switzerland) was placed loosely around the pouch. Non-absorbable sutures were used to fix the ring to the stomach passing through the built-in holes in the ring. Concomitant operative procedures included crural repair when hiatal hernia was present using unidirectional barbed 2/0 V-Loc non-absorbable sutures (Covidien, Mansfield, MA, USA), and cholecystectomy using the same ports.

### Inclusion and Exclusion Criteria

The inclusion criteria for this study were patients who had undergone primary laparoscopic sleeve gastrectomy and had experienced weight regain exceeding their nadir, as reported by those with a body mass index (BMI) of 35 kg/m^3^ or higher. Furthermore, an age between 18 and 60 years [20, 21]. In addition, the stomach dilation volume during MDCT was defined as either 250 mL or greater of the total stomach volume and/or the presence of the fundus. Patients meeting these criteria were included in the study [8, 13].

The exclusion criteria were the presence of gastroesophageal reflux disease (GERD) according to the Los Angeles classification, diagnosed by routine pre-operative gastroscopy (EGD), [22], or patients’ preference for malabsorptive surgery.

### Data Collection

Preoperative data, such as patient’s demographics, BMI, the time between the primary and revision procedures, associated medical problems, EGD and imaging findings, sleeve volume assessed by MDCT virtual gastroscopy, and laboratory investigations were collected.

Perioperative data included operation time and combined operative procedures, such as hiatal hernia repair and cholecystectomy.

Postoperative data included early and late complications, nutritional laboratory results, EGD for diagnosing GERD de novo or hiatal hernia, result changes in BMI, %EWL, %TWL, and improvement of associated medical problems at one and two-year follow-ups after revision surgery.

### Preoperative Care

A multidisciplinary team (MDT) evaluation included a bariatric metabolic surgeon, a dietician, an internist, an outpatient clinic nurse, and a psychiatrist, who all worked up the patient prior to surgery.

### Postoperative Care

Prophylaxis against venous thrombosis was started 12 h before surgery with enoxaparin and was continued for 21 days after surgery. A routine gastrografin swallow was performed on day one after surgery. MDCT with intravenous (IV) and oral contrast was performed when patients had alarming symptoms, such as tachycardia, persistent abdominal pain, fever, persistent vomiting, and abdominal distension. Multi-vitamins, calcium, and iron supplements were prescribed to all patients.

### Statistical Analysis

For the analyses, we used descriptive and inferential statistics. All data were first tested for normality using the Kolmogorov-Smirnov test, Q-Q plot, and Levene’s test. Categorical variables were expressed as n (%). Continuous normally distributed variables were expressed with their means and standard deviations, while non-normally distributed variables were expressed with their medians and interquartile ranges. Categorical variables were tested using Pearson’s chi-squared test or Fisher’s exact test, when appropriate. Continuous normally distributed data were tested with the Student’s *t*-test for independent samples. For non-normally distributed data, the Mann-Whitney *U*-test was used for independent samples. Statistical significance was set at *p* ≤ 0.05. Statistical analyses were performed using the R software (version 4.1.3. package)

### Sample Size

The sample size was calculated using G*power Version 3.1.9.5, based on a large effect size of 0.8, corresponding to a mean difference in %EWL two years after re-sleeve gastrectomy of at least 8% (±sd 10) between BLSG and NBLSG with a power of 80% and a significance level of 0.05. This resulted in a minimum required sample size of 26 patients per group. Considering a possible loss of patients to follow-up, an additional 10% increase in sample size was included, resulting in a minimum of 57 patients enrolled.

### Randomization

A single-blinded randomization procedure was performed in which patients and outpatient clinic nurses were blinded for the whole study period. The surgeon was informed of the allocation after the patient was under anesthesia. Randomized block randomization was performed using computer-generated blocks of two or four block sizes. When the patient was lost to follow-up, the therapy allocation was explained by a letter, and the patient could always contact the clinic.

### Data Capture

The analysis was performed on a blinded dataset after the completion of the medical/scientific review. All protocol violations were identified and resolved, and the dataset was declared complete. All data were collected in a data management system (Castor EDC, Amsterdam, The Netherlands; https://www.castoredc.com) and handled according to Good Clinical Practice guidelines, Data Protection Directive certificate, and complied with Title 21 CFR Part 11. Furthermore, the data centers, where all the research data were stored, were certified according to ISO27001, ISO9001, and Dutch NEN7510.

## Results

### Baseline Characteristics

This single blinded-randomized controlled trial included 82 patients referred from other centers. Four patients were excluded for being older than 60 years, ten for suffering from GERD, seven for refusing a re-LSG procedure, and two for a history of a previous leak after LSG.

**Fig. 1:**
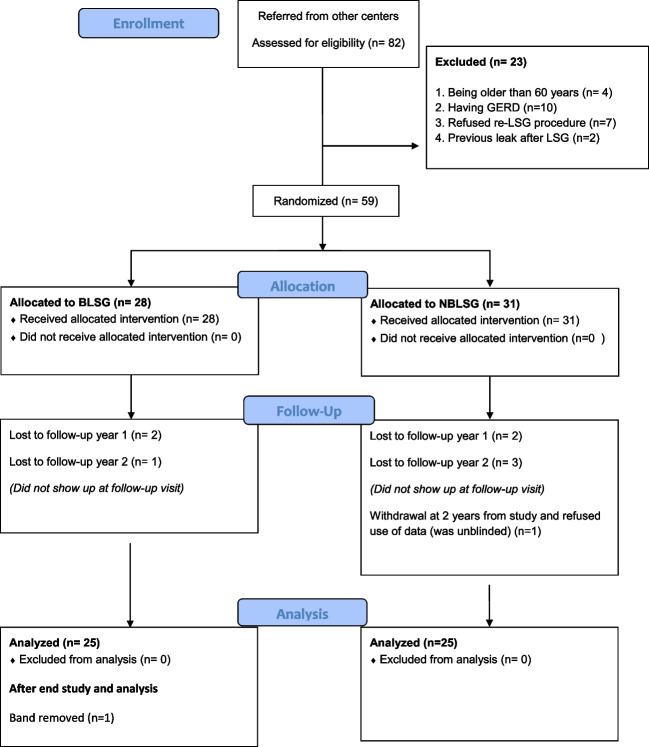
CONSORT 2010 flow diagram

After one year, two patients were lost to follow-up in the BLSG group and two in the NBLSG group; in year two, 25 patients were left in each group (-1 in BLSG and -4 in NBLSG), and this was the total number of patients analyzed (CONSORT flow diagram, Fig. 1) [23].

There were no significant differences in baseline characteristics, including demographic data like age (43.0 ± 7.2 years), BMI before first and second surgeries in BLSG and NBLSG groups (51.8 vs. 51.4 kg/m^2^, and 47.7 vs. 46.9 kg/m^2^, respectively), associated medical problems, the time between first LSG and re-LSG (3.8±0.8 years), smoking status (28 vs. 40%), laboratory results, preoperative stomach volume (408 vs. 410 ml), food tolerance (22.9±0.8), or endoscopic findings before re-sleeve (4.0 vs. 8.0% with hiatal hernia) (Table 1).

**Table 1:** Baseline characteristics of patients in banded and non-banded re-sleeve gastrectomy groups

Baseline characteristic	Banded re-sleeve (BLSG) N = 25	Non-banded re-sleeve (NBLSG) N = 25	*P* value
Age (years), mean ± SD	40.7 ± 11.2	39.7 ± 8.0	.707
Sex (female), n (%)	21 (84.0%)	17 (68.0%)	.321
Anthropometrics, mean ± SD			
Height (m)	1.7 ± 0.1	1.7 ± 0.1	.787
Weight before 1^st^ surgery (Kg)	141.8 ± 18.7	141.2 ± 15.3	.908
BMI before 1^st^ surgery (Kg/m^2^)	51.8 ± 4.5	51.4 ± 5.8	.804
Weight before 2^nd^ surgery (Kg)	131.0 ± 21.1	129.9 ± 24.2	.863
BMI before 2^nd^ surgery (Kg/m^2^)	47.7 ± 5.0	46.9 ± 6.1	.589
The time between first sleeve and re-sleeve (years), mean ± SD	3.8 ± 0.8	3.8 ± 0.8	.862
Smoking, n (%)	7 (28.0%)	10 (40.0%)	.551
Associated medical problems before the first sleeve, n (%)			
Hypertension	8 (32.0%)	8 (32.0%)	1.000
Diabetes	4 (16.0%)	6 (24.0%)	.725
Dyslipidemia	7 (28.0%)	8 (32.0%)	1.000
Pre-re-sleeve lab investigations, mean ± SD			
HG	13.2 ± 1.6	13.0 ± 1.8	.661
WBC	7.3 ± 2.3	7.4 ± 2.2	.775
SGOT	22.1 ± 7.6	20.8 ± 9.7	.618
SGPT	32.0 ± 12.3	29.0 ± 11.6	.378
UREA	26.3 ± 8.2	26.9 ± 6.5	.777
CRT	0.8 ± 0.3	0.8 ± 0.2	.682
INR	1.1 ± 0.1	1.0 ± 0.1	.499
T3	2.8 ± 1.0	3.1 ± 0.7	.269
T4	1.2 ± 0.5	1.3 ± 0.5	.515
TSH	2.2 ± 1.5	2.9 ± 1.1	.055
FBS	100.4 ± 24.9	109.4 ± 33.1	.281
HbA1c	7.7 ± 1.5	8.0 ± 1.7	.458
Preoperative stomach volume (ml), mean ± SD	408.0 ± 74.6	410.0 ± 66.1	.921
Preoperative food tolerance	22.9 ± 0.8	22.9 ± 0.8	1.000
Endoscopy before re-sleeve, n (%)			
Free	24 (96.0%)	23 (92.0%)	1.000
Hiatal hernia	1 (4.0%)	2 (8.0%)	

*HG*, hemoglobin; *WBC*, white blood cells; *SGOT*, serum glutamic-oxaloacetic transaminase; *SGPT*, serum glutamic-pyruvic transaminase; *CRT*, creatinine; *INR*, international normalized ratio; *TSH*, thyroid stimulating hormone; *FBS*, fasting blood sugar; *HbA1c*, glycated hemoglobin; *SD*, standard deviation

### Perioperative

There was no significant difference in the operative time between the BLSG and NBLSG groups (101.6 vs. 99.1 min, respectively; *p* = .498). Both groups had combined surgery during the re-LSG; 20.0% in the BLSG group and 16.7% in the NBLSG group had chronic calcular cholecystitis, for which a cholecystectomy was performed. In 4% of the BLSG group and 8% of the NBLSG group, a hiatal hernia detected preoperatively by endoscopy was repaired. Another hiatal hernia in the BLSG group (4%) and two (8%) in the NBLSG group were found intraoperatively. In the other 4.0%, both BLSG and NBLSG had a combination of cholecystectomy and hiatal hernia performed during the re-LSG (*p*=1.00) (Table 2).

**Table 2:** Operative and postoperative outcomes in banded and non-banded re-sleeve gastrectomy groups

	Banded re-sleeve (BLSG) N = 25	Non-banded re-sleeve (NBLSG) N = 25	*P* value
Operative time (min), mean ± SD	101.6 ± 111.6	99.1 ± 14.7	.498
Combined surgery, n (%)			1.000
CCC*	5 (20.0%)	4 (16.7%)	
Hiatal hernia	2 (8.0%)	4 (16.0%)	
CCC + hiatal hernia	1 (4.0%)	1 (4.0%)	
BMI after re-sleeve, mean ± SD			
At six months	36.3 ± 3.9	36.5 ± 4.1	.838
At year 1	26.9 ± 1.9	26.3 ± 1.5	.234
At year 2	24.9 ± 2.2	26.0 ± 1.6	**.045**
Excess weight loss (%EWL), mean ± SD			
At six months	46.9±8.4	43.6±10.1	.259
At year 1	83.7±9.9	86.3±4.7	.151
At year 2	85.7±9.6	83.9±4.9	.199
Total weight loss (%TWL), mean ± SD			
At six months	23.9±4.4	21.8±5.8	0.342
At year 1	43.1±7.7	43.3±6.1	0.497
At year 2	44.2±8.1	42.2±5.9	0.891
Food tolerance, mean ± SD			
After year 1	21.1 ± 0.8	21.2 ± 0.8	.601
After year 2	20.3 ± 0.8	21.4 ± 0.5	**< .001**
Stomach volume (mL), mean ± SD			
After year two postoperative	159.6 ± 7.6	194.2 ± 10.7	**< .001**
Year 2 volume above the band	72.4 ± 16.5		
Improved associated medical problems after the first sleeve			
Hypertension	6 (75.0%)	5 (62.5%)	1.000
Diabetes	2 (50.0%)	3 (50.0%)	1.000
Dyslipidemia	3 (42.9%)	4 (50.0%)	1.000
Improved associated medical problems two years after the second sleeve			
Hypertension	8 (100.0%)	8 (100.0%)	1.000
Diabetes	4 (100.0%)	6 (100.0%)	1.000
Dyslipidemia	5 (71.4%)	6 (75.0%)	1.000

*CCC, chronic calcular cholecystitis; *SD*, standard deviation

The numbers in bold is the statistical significance (*p*<0.05)

### Postoperative

#### ΔBMI, %EWL, %TWL

The two re-LSG groups achieved similar % EWL and %TWL at six months, one year, and two years postoperatively (%EWL 46.9 vs. 43.6, 83.7 vs. 86.3, and 85.7 vs. 83.9) (*p*= > 0.151) (%TWL 23.9 vs.21.8, 43.1 vs.43.3, 44.2 vs. 42.2) (*p*=>0.342).

**Fig. 2:**
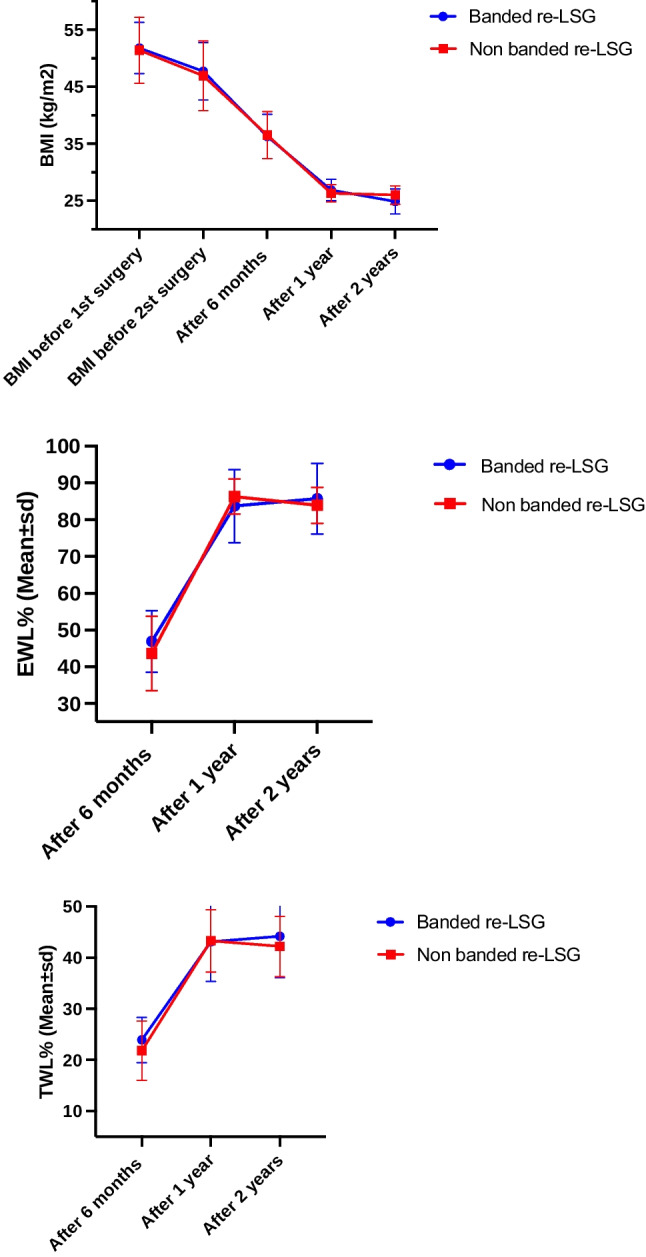
Body mass index changes, excess weight loss percentage, and total weight loss percentages after banded and non-banded re-sleeve gastrectomy

Only the BMI after re-LSG was significantly lower at two years in the BLSG group (24.9 vs. 26.9 in the NBLSG group [*p* = 0.045]; Fig. 2, Table 2).

Furthermore, postoperatively between years one and two, there was a significant difference between both groups; in the NBLSG, 20 patients (80%) had a 2 kg increase in weight (0.5–5 kg). Whereby in the BLSG, in only six patients (24.0%), their weight increased at the two-year point. Median (min-max) of 1 kilogram (kg) (1–5 kg) (*p*=0.001).

Overall, the BMI was significantly reduced two years postoperatively in both groups compared to pre-revisional surgery (47.7 to 24.9 in BLSG and 46.9 to 26.0 kg/m in NBLSG; *p* = <0.001).

#### Food Tolerance

**Fig. 3:**
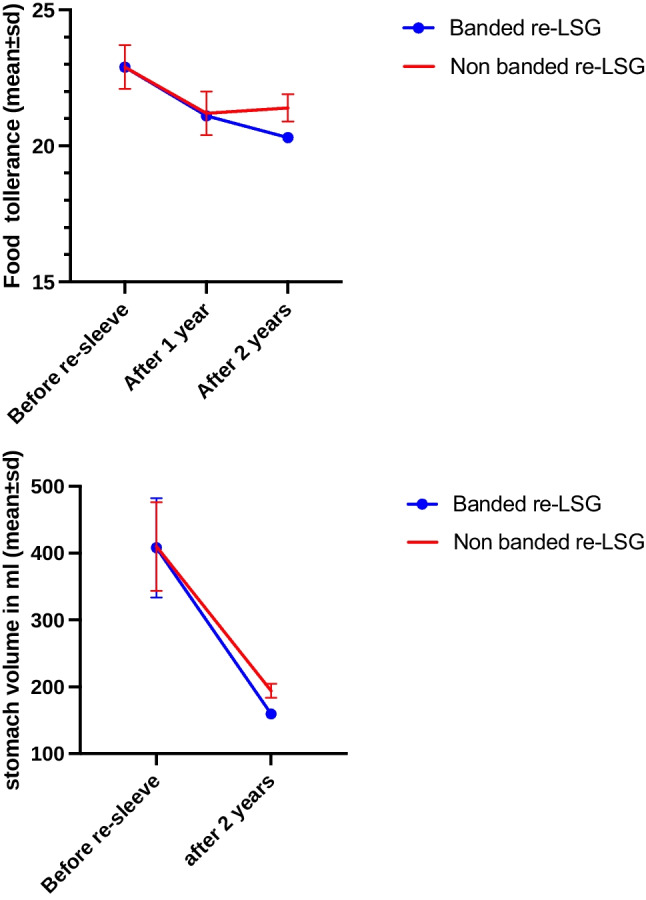
Food tolerance and whole stomach volume after banded and non-banded resleeve gastrectomy

Food tolerance after two years was significantly lower with BLSG; therefore, the BLSG group had a worse outcome than NBLSG with an average of -1.1 points (95% confidence interval [CI]: 0.7 to 1.5; *p*= < 0.001). Nevertheless, the reduced postoperative stomach volume significantly reduced the food tolerance score in both groups (*p*= < 0.001; Table 2; Fig. 3).

#### Stomach Volume

The new stomach volume was significantly smaller in the BLSG group compared to the NBLSG group by 34.6 mL on average (159.6 vs. 194.2 mL, *p*=<0.001), and both groups showed a significant reduction in volume after two years (-248.4 mL vs. -215.8 mL [*p*=<0.001) in the BLSG and NBLSG groups, respectively).

**Fig. 4:**
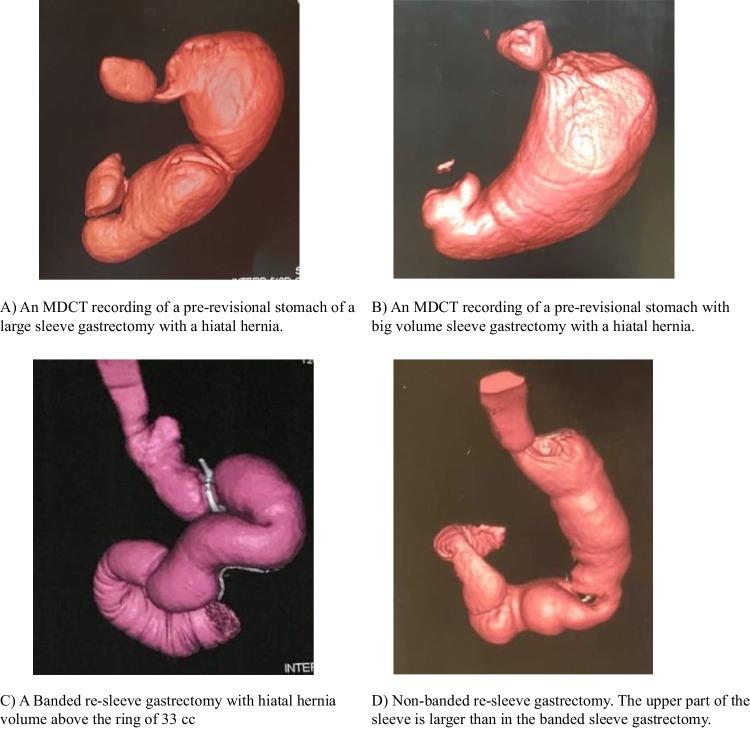
Multi-detector computed tomography (MDCT) imaging of pre- and postrevision sleeve gastrectomy

After two years, the volume above the band in the BLSG was 72.4±16.5 mL (Table 2, Figs. 3 and 4).

#### Associated Medical Problems

Improvement of the associated medical problems one and two years after the second LSG was significantly lower, but no significant differences between both groups. Hypertension significantly improved in 75.0% and 62.5% of patients after the first sleeve in the BLSG and NBLSG groups, respectively, and in 100% after the second sleeve within both groups.

Diabetes significantly improved by 50.0% after the first sleeve procedure and 100% after the second sleeve within both groups.

Finally, dyslipidemia significantly improved after the first sleeve and second sleeve in both groups (42.9% in BLSG, 50.0% in NBLSG, versus 71.4% in BLSG and 75.0% in NBLSG, respectively; Table 2).

#### Complications

Postoperative complications were not significantly different between BLSG and NBLSG (84.0 vs. 88.0%, *p*=1.00). Three patients in each group (12.0%) developed chronic calcular cholecystitis, and one in the BLSG group developed a port site hernia (4.0%). No cases of intrathoracic sleeve migration of the stomach was noted.

One patient (4%, *p*=0.784) in the BLSG group had the band removed after two years at the end of the study because of persistent complaints of GERD C and dysphagia and was converted to an RYGB operation.

No leaks, abscesses, 30-day post-op interventions, band erosions, or band slippages were reported during the study period.

#### Endoscopic Findings

After one year, during the endoscopic procedure, 28.0% in the BLSG group and 24.0% in the NBLSG group were free from complications. However, 8.0% of the BLSG group had constriction at the ringside, and 48.0 vs. 60.0% had asymptomatic GERD A de Novo in the BLSG and NBLSG groups, respectively. Moreover, 4% had GERD B de Novo in the BLSG group, compared to 0% in the NBLSG group.

GERD B + hiatal hernia de Novo was present in 8.0% of the BLSG and 16.0% of the NBLSG groups. One patient (4.0%) in the BLSG developed a hiatal hernia de Novo postoperatively.

After two years, endoscopy was performed on indication in three patients in the BLSG group and two in the NBLSG group, with no significant difference between the findings in both groups (Table 3). In the BLSG group, one patient (33.3%) was free of any diagnoses, one (33.3%) had GERD C+ hiatal hernia de Novo, and one (33.3%) had hiatal hernia de Novo. In the NBLSG group, two patients (100%) had GERD C+ hiatal hernia de Novo.

**Table 3:** Postoperative complications, endoscopic findings, and nutritional deficiencies two years after the re-sleeve gastrectomy

	Banded re-sleeve (BLSG) N = 25	Non-banded re-sleeve (NBLSG) N = 25	*P* value
Postoperative complications, n (%)			1.000
None	21 (84.0%)	22 (88.0%)	
CCC*	3 (12.0%)	3 (12.0%)	
Port site hernia	1 (4.0%)	0 (0.0%)	
Dysphagia	1 (4.0%)	-	
Routine endoscopy after one year, n (%)			.500
Free	7 (28.0%)	6 (24.0%)	
Constriction at ring	2 (8.0%)	0 (0.0%)	
GERD A (asymptomatic)	12 (48.0%)	15 (60.0%)	
GERD B	1 (4.0%)	0 (0.0%)	
GERD B, hiatal hernia	2 (8.0%)	4 (16.0%)	
hiatal hernia	1 (4.0%)	0 (0.0%)	
Endoscopy after two years (when indicated), n (%)	n = 3	n = 2	1.000
Free	1 (33.3%)	0 (0.0%)	
GERD C, hiatal hernia	1 (33.3%)	2 (100.0%)	
Hiatal hernia	1 (33.3%)	0 (0.0%)	
Nutritional deficiencies after two years, n (%)			
Ferritin < 30	4 (16.0%)	0 (0.0%)	.110
Calcium < 8.6	7 (28.0%)	12 (48.0%)	.244
Vitamin D < 20	6 (24.0%)	5 (20.0%)	1.000
Vitamin B12 < 200	1 (4.0%)	3 (12.0%)	.609
Albumin < 3	4 (16.0%)	2 (8.0%)	.667
Hemoglobin < 11	10 (40.0%)	9 (36.0%)	1.000

*CCC, chronic calcular cholecystitis; *GERD*, gastroesophageal reflux disease

#### Nutritional Deficiencies

After two years, no significant differences in nutritional deficiencies (*p* ≥ 0.110) were found between both groups. The most prevalent deficiencies were calcium < 8.6 mg/dL (28.0% in the BLSG group and 48.0% in the NBLSG group), vitamin D < 20 ng/mL (24.0 vs 20.0%), and hemoglobin < 11 mg/dL (40.0 vs 36.0%).

## Discussion

This single-blinded randomized controlled trial compared the outcomes of revisional BLSG and NBLSG throughout two years of follow-up. Both BLSG and NBLSG had comparable significant weight loss effects and improvement of associated medical problems in patients. At our clinic, we provide comprehensive explanations of various bariatric metabolic revisional surgeries to all patients. Following a thorough consultation with the multidisciplinary team (MDT) regarding eligibility criteria and optimal surgical options, we present the recommended procedures to our patients. However, we have increasingly encountered patients who prefer LSG as their sole choice for revisional surgery after experiencing insufficient weight loss or weight regain, accompanied by an enlarged stomach volume. Consequently, we decided to further investigate this growing trend.

### Stomach Volume and BMI

In our study, the preoperative stomach volume was increased after undergoing primary LSG, which might have contributed to weight regain in these patients. This was confirmed by Weiner et al. [24], measured the total gastric volume before and the removed gastric volume after LSG in three groups ( removed volume: 490.2 mL, 732.7 mL, and 1156.1 mL); in the follow-up periods, they concluded that removal of less than 500 mL of gastric volume might result in early weight regain after LSG. Furthermore, Disse et al. found that sleeve dilatation was present in more than 50% of patients after LSG; however, this dilatation was not linked to increased daily caloric intake or insufficient weight loss during the first 18 months following surgery [8]. This was confirmed by another study that reported that sleeve volumes almost doubled within the first post-operative year [25].

Moreover, Brachetto et al. reported that the mean capacity of the stomach increased from 108±25 on the third operative day to 250± 85 ml in the follow-up period (*p*= 0.0001) in patients who underwent LSG; however, this was not correlated with weight regain [26].

Thus, we may assume that an average postoperative volume of 108 mL with a two-fold increase after 24–36 months does not cause any weight to regain [8, 26]. However, in our study, patients had a four-fold stomach volume, which might have contributed to the weight regain since more dilation is correlated to less restriction and, therefore, more food consumption. A study by Hany et al. found that in a retrospective trial with four years of follow-up after primary LSG with or without a ring, that banded LSG had significantly lower sleeve volume, significantly lower WR, and significantly lower FT scores than LSG and that the ring can work as a possible prevention against stomach enlargement [27].

We aim to find solutions and test this scientifically to create the best possible option for the patients: (1) In the solution for weight loss and reduction of associated medical problems (primary and revision surgery in general) and (2) the solution is to prevent weight regain (placement of a band, adherence to lifestyle changes, behavior and eating treatment, and assessment of psychological factors) and try to stabilize the patient’s health over time.

So, when patients have postoperative weight regain after LSG, an MDCT test can help determine whether dilation of the stomach has occurred in combination with weight regain. Therefore, attention to eating habits, psychological factors, and hormonal status is essential in assessing weight loss failure despite the absence of a significant increase in stomach volume. In our study, it was difficult to correlate the reasons for weight regain since the preoperative stomach volume from the first LSG operation was unknown.

A possible explanation for weight gain in patients who underwent primary LSG that those with dilated LSGs may have had a smaller total gastric volume at baseline. This could result in more significant changes in sleeve dilatation when the sleeve what was created was too narrow [8]. Furthermore, weight regain without dilatation of the stomach volume may occur in patients who received an LSG with excessive gastric tube or antrum remaining, resulting in reduced weight loss and earlier weight regain [24].

After two years, there was a significant reduction in stomach volume and BMI in the BLSG and NBLSG groups. Furthermore, after two years, the extra BMI loss was in the BLSG group with a smaller stomach volume. The literature shows equal initial weight loss between the LBSG and the NBLSG or better weight loss in the BLSG [14, 17, 18, 28-31].

A study by Filip et al., who followed up patients for three years, also reported a reduction in stomach volume and BMI after re-LSG [13].

Furthermore, this RCT showed that the %EWL and %TWL in the NBLSG slightly decreased, and weight increased in 80% of the NBLSG patients (24% in the BLSG) after two years of follow-up with significant differences between both groups on the weight increase. Thus, band placement may help reduce stomach volume and prevents weight increase after two years for better postoperatively results. Longer follow-up must prove the related effects of the band in the years to come on the clinical implication after revisional BMS with and without a band placement.

Our study focused on only the increased volume of the stomach within patients who were stable and compliant in lifestyle and behavior instead of other causes of weight regain of insufficient weight loss. We used a strict inclusion criterion for this study, that in an MDT, every patient was screened and discussed. All had a pre-diet plan, assessment by a psychiatrist, and checked on lifestyle factors. So, we had a clear sample with low bias or confounding factors.

### Revisional Surgery

It is worth noting that several options for managing weight regain after failed LSG are available for patients who are not eligible for re-LSG because of GERD or for other medical reasons, or who refuse a re-LSG. In a 2014 systematic review, RYGB after LSG, and other surgical interventions performed after LSG was reported to achieve a %EWL of 60 and 48 % over 12 and 24 months, respectively [32]. Moreover, a 2022 randomized controlled trial reported that revisional RYGB and one-anastomosis gastric bypass had comparable significant weight loss effects after failed LSG with % excessive BMI loss of 89.3±15.4 and 84.8±18.2%, respectively, after two years; both procedures were safe with no significant differences in complications and nutritional deficits [33]. Multiple systematic reviews and studies that tested alternative revision surgeries after (failed)-LSG confirmed the same result [31, 34-40]. Therefore, consultation with the patient and a multi-disciplinary team on the choice of revision surgery is essential, whereby all interventions achieve good health-related benefits.

### Food Tolerance

Both LBSG and NBLSG groups showed a significantly reduced food tolerance (FT) score two years postoperatively, which may be explained by the reduced stomach volume. The BLSG group showed a significantly lower score than the NBLSG group, probably caused by the restrictive feeling from the band over time. Banded procedures generally have lower FT scores than non-banded procedures, such as laparoscopic adjustable gastric banding, which has the worst reported FT among bariatric metabolic surgery procedures [41]. Moreover, the reported FT was significantly lower in banded-RYGB compared to non-banded RYGB [42]. However, no evidence correlates a higher FT score to weight regain.

In several studies, the banded sleeve had a higher incidence of regurgitation, vomiting, and dysphagia, reflected in a lower FT score [14, 16, 18]. Moreover, our study had one patient (4%) who had the band removed after two years after persistent complaints of GERD C and complications of dysphagia. The patient was unblinded to provide additional care. The symptoms improved after the removal of the band and conversion to the RYGB procedure. A 2019 systematic review on a small number of cases found complication rates of 11.8%, and reoperation rates of 5.5% following BLSG [12], which align with our reoperation rates. Thus, good instructions and preparation are necessary, especially for BLSG, since the patient may experience discomfort after the revision LSG procedure.

### Complications

Asymptomatic GERD A de novo significantly increased postoperatively after one year and decreased after two years. This higher incidence and the coincidental finding of asymptomatic GERD A during the EGD can be explained by the fact that the new stomach had more restrictions with significantly lower volume after a long period of the dilating stomach. Furthermore, the incidence of GERD was slightly but insignificantly lower in the BLSG group. A study by Fink et al. also reported having lower rates of GERD with BLSG compared to NBLSG [18]. The anti-reflux mechanism could be correlated to the mechanical prevention of the reflux of gastric fluids into the esophagus by the band. At the same time, the part of the stomach above the ring has few acid-secreting glands. However, our study lacks sufficient power to provide evidence that would assist decision-making on the most profitable operation to avoid reflux. A study on BLSG and NBLSG found GERD in 109 patients (16.6%) in the NBLSG group vs. 19 patients (14.4%) in the BLSG group, with no significant difference between them (*p* = 0.552). Whereby no patients needed a revision operation because of GERD in the BLSG group, 43 (6.5%) patients who underwent NBLSG needed an operation. In the same study, 25 patients presented with esophagitis preoperatively in the BLSG group, and with postoperative routine endoscopy, a regression of reflux in 21 (84%) was noticed. So far, substantial evidence supports the higher possibility that Barrett's esophagus (BE) specific to the banded sleeve is missing. Nevertheless, when looking at specific LSG without the band, a very recent study by Wölnerhanssen et al. from 2023 studied at least 5 years of follow-up data. This study found a higher incidence of reflux symptoms, reflux-esophagitis, and pathological esophageal acid exposure in LSG- compared to RYGB patients. However, the incidence of BE after LSG was low and not significantly different between groups [43].

Still, this study showed that after re-LSG, a lower GERD A in the BLSG (12 vs. 15 in the NBLSG) and lower grade B (3 vs. 4 in the NBLSG) were found during the routine endoscopy, wherefore the band can help prevent GERD after LSG over time. So banded procedures could have beneficial effects in preventing GERD and reflux symptoms and have a lower incidence of re-operation than an LSG procedure, as reported by Hany et al. [28]. A more comprehensive study would be needed to continue to follow the potential long-term complications.

### Limitations

This study had some limitations, such as the lack of data on the first operation or the postoperative stomach volume at three months since all the patients were referred from other clinics. For example, calibrations of the LSG procedure, how the follow-up was done, guidance and therapy from the multi-disciplinary team, and how the weight loss in that period went were unknown. Moreover, the cause of either primary or secondary sleeve pouch dilatation was unclear, even with the MDCT volumetry, so we did not correlate this data with the weight loss before and after the re-LSG. Another limitation is the relatively short 2-year follow-up time, which may be insufficient for a proper conclusion of outcomes between the two groups. At the same time, the differences after three or more years could be significant in favor of one of the groups. Moreover, nine of 59 patients were lost to follow-up. This may be explained by a lack of interest in follow-up after successful revision surgery, mainly since the patients were referred from other centers. We did not measure any quality of life questionnaires pre-revision and follow-up. This could have been with added value and must be included in new studies as an outcome measure.

We recommend studying the effect of successful or failed LSG on stomach volume in future cohorts, focusing on the cut-off values for dilatation vs. volume vs. weight regain. Finally, this study was powered to detect only a large effect size corresponding to approximately 8% differences in %EWL between both re-sleeve groups; if there are medium or minor differences in %EWL between the two re-sleeve groups, a larger sample would be required to detect such differences.

## Conclusion

Laparoscopic re-LSG is feasible and safe with satisfactory outcomes in patients with weight regain after LSG who have gastric dilatation without reflux esophagitis. Both BLSG and NBLSG had comparable significant weight loss effects when performed as re-LSG operations with a comparable improvement of associated medical problems. The BLSG tends to have a more stable weight loss after two years with a significantly lower BMI, lower stomach volume, and less weight regain. Food tolerance was decreased in both groups but more reduced in the BLSG group. After a 2-year follow-up, both procedures were considered safe, with no significant differences in the occurrence of complications and nutritional deficits.

## Appendix 1

See Table 4.

**Table 4:** CONSORT 2010 checklist of information to include when reporting a randomised trial*

Section/topic	Item No	Checklist item	Reported on page No
Title and abstract			
	1a	Identification as a randomised trial in the title	Page 1
	1b	Structured summary of trial design, methods, results, and conclusions (for specific guidance see CONSORT for abstracts)	Page 1
Introduction			
Background and objectives	2a	Scientific background and explanation of rationale	Page 3-4
	2b	Specific objectives or hypotheses	Page 3-4
Methods			
Trial design	3a	Description of trial design (such as parallel, factorial) including allocation ratio	Page 5
	3b	Important changes to methods after trial commencement (such as eligibility criteria), with reasons	Page 5
Participants	4a	Eligibility criteria for participants	Page 5-6
	4b	Settings and locations where the data were collected	Page 5
Interventions	5	The interventions for each group with sufficient details to allow replication, including how and when they were actually administered	Page 7-8
Outcomes	6a	Completely defined pre-specified primary and secondary outcome measures, including how and when they were assessed	Page 6-7
	6b	Any changes to trial outcomes after the trial commenced, with reasons	Page 6-7
Sample size	7a	How sample size was determined	Page 9
	7b	When applicable, explanation of any interim analyses and stopping guidelines	n.a.
Randomisation:			
Sequence generation	8a	Method used to generate the random allocation sequence	Page 9
	8b	Type of randomisation; details of any restriction (such as blocking and block size)	Page 9
Allocation concealment mechanism	9	Mechanism used to implement the random allocation sequence (such as sequentially numbered containers), describing any steps taken to conceal the sequence until interventions were assigned	Page 9
Implementation	10	Who generated the random allocation sequence, who enrolled participants, and who assigned participants to interventions	Page 9
Blinding	11a	If done, who was blinded after assignment to interventions (for example, participants, care providers, those assessing outcomes) and how	Page 9
	11b	If relevant, description of the similarity of interventions	Page 9
Statistical methods	12a	Statistical methods used to compare groups for primary and secondary outcomes	Page 9
	12b	Methods for additional analyses, such as subgroup analyses and adjusted analyses	Page 9
Results			
Participant flow (a diagram is strongly recommended)	13a	For each group, the numbers of participants who were randomly assigned, received intended treatment, and were analysed for the primary outcome	Page 10
	13b	For each group, losses and exclusions after randomisation, together with reasons	Page 10
Recruitment	14a	Dates defining the periods of recruitment and follow-up	Page 10
	14b	Why the trial ended or was stopped	n.a.
Baseline data	15	A table showing baseline demographic and clinical characteristics for each group	Page 10
Numbers analysed	16	For each group, number of participants (denominator) included in each analysis and whether the analysis was by original assigned groups	Page 10-14
Outcomes and estimation	17a	For each primary and secondary outcome, results for each group, and the estimated effect size and its precision (such as 95% confidence interval)	Page 10-14
	17b	For binary outcomes, presentation of both absolute and relative effect sizes is recommended	Page 110-14
Ancillary analyses	18	Results of any other analyses performed, including subgroup analyses and adjusted analyses, distinguishing pre-specified from exploratory	Page 10-14
Harms	19	All important harms or unintended effects in each group (for specific guidance see CONSORT for harms)	Page 10-14
Discussion			
Limitations	20	Trial limitations, addressing sources of potential bias, imprecision, and, if relevant, multiplicity of analyses	Page 15-19
Generalisability	21	Generalisability (external validity, applicability) of the trial findings	Page 15-19
Interpretation	22	Interpretation consistent with results, balancing benefits and harms, and considering other relevant evidence	Page 15-19
Other information			
Registration	23	Registration number and name of trial registry	Page 5
Protocol	24	Where the full trial protocol can be accessed, if available	Title page
Funding	25	Sources of funding and other support (such as supply of drugs), role of funders	Page 20

*We strongly recommend reading this statement in conjunction with the CONSORT 2010 Explanation and Elaboration for important clarifications on all the items. If relevant, we also recommend reading CONSORT extensions for cluster randomised trials, non-inferiority and equivalence trials, non-pharmacological treatments, herbal interventions, and pragmatic trials. Additional extensions are forthcoming: for those and for up to date references relevant to this checklist, see www.consort-statement.org

## Appendix 2

### Multi-detector computed tomography (MDCT)

Image acquisition was performed in the spine position and limited to the stomach, which is adequately inflated with gas on the topogram. Scans were acquired using the least radiation dose with the following parameters: 80 KV, 125 mA, 32 × 0.6 mm collimation, with 1 mm slice thickness reconstruction using SAFIRE iterative reconstruction. Data were transferred to a dedicated 3D workstation. Three-dimensional volume-rendering images were created by a combination of manual and semi-automatic segmentation tools. Different masks were created to represent the various relevant structures in different colors. The volume of the stomach (in preoperative series) and the sleeve pouch was measured on multiplanar reformations. Volume of the resected stomach was estimated by subtracting the pouch volume at 6 months postoperatively from the preoperative stomach volume, putting in consideration that intraoperative sleeve pouch construction was standardized throughout the study. The whole procedure was performed and interpreted in all patients by one radiologist
